# Whole-genome sequencing-based source tracing and infection control of *Serratia marcescen*s blood culture events in pediatric patients

**DOI:** 10.3389/fmicb.2025.1718340

**Published:** 2026-01-16

**Authors:** Wang Zhang, Caihua Ma, Falin Xu, Chenjing Zhao, Ling Wang

**Affiliations:** 1 Department of Infection Prevention and Control, The Third Affiliated Hospital of Zhengzhou University, Zhengzhou, China; 2 Department of Pediatrics, The Third Affiliated Hospital of Zhengzhou University, Zhengzhou, China

**Keywords:** infection control, infection control (ICP) practitioners, sepsis, *Serratia marcescens*, whole genome sequencing

## Abstract

**Objective:**

*Serratia marcescen*s (*S. marcescens*) is an opportunistic pathogen increasingly associated with nosocomial infections in immunocompromised pediatric patients. This study aimed to investigate the epidemiological and molecular epidemiological characteristics of *S. marcescens*-positive blood cultures and to provide evidence for targeted infection prevention strategies.

**Methods:**

Eleven cases of *S. marcescens*-positive blood cultures were identified across eight pediatric wards of a tertiary hospital in China in October 2024. Clinical and microbiological assessments were conducted to distinguish true infections from contamination. Antimicrobial susceptibility testing was performed, and whole-genome sequencing (WGS) was used to characterize resistance, virulence, and plasmid replicons. Core genome single-nucleotide polymorphism (SNP)-based phylogenies, Bayesian temporal inference, and transmission tree reconstruction were used to explore genetic relatedness, transmission dynamics, and cryptic cases.

**Results:**

Two cases were diagnosed as hospital-acquired sepsis, while nine were classified as contamination. The isolates exhibited intrinsic resistance to first- and second-generation cephalosporins but remained susceptible to carbapenems. Seven resistance genes—including aac(6’)-Ic, oqxB, and tet(41)—and four virulence genes, including cheY and fliM, were identified. Nine isolates carried IncFII-type plasmids. Core genome SNP analysis revealed minimal genetic divergence, with the most recent common ancestor traceable to late 2023, suggesting approximately 10 months of silent transmission. Transmission tree inference further indicated the presence of undetected cryptic cases. Following the implementation of bundled interventions—including sink replacements and disinfectant use, reinforcement of hand hygiene, and environmental decontamination—no new cases occurred during 1 month of follow-up.

**Conclusion:**

This study identified a highly clonal *S. marcescens* cluster with evidence of prolonged, unnoticed circulation and cross-ward transmission in the pediatric units. These findings underscore the hidden persistence of this pathogen in the hospital environment and the need for strengthened infection control measures.

## Introduction

*Serratia marcescens* is a Gram-negative, facultatively anaerobic bacillus commonly found in natural environments and in moist areas of healthcare facilities, such as faucets, wet towels, and various medical devices (Bourdin et al., 2023). Although it exhibits limited pathogenicity in immunocompetent individuals, it can cause severe healthcare-associated infections, including bloodstream infections, pneumonia, and urinary tract infections, in neonates, critically ill patients, and individuals receiving prolonged invasive procedures (Crivaro et al., 2007). In recent years, *S. marcescens* has gained increasing clinical attention due to its multidrug resistance and diverse virulence factors, with particularly elevated transmission risk in pediatric and neonatal wards (Reddy et al., 2021; Arslan et al., 2010). The organism frequently shows resistance to cephalosporins, aminoglycosides, and fluoroquinolones (Martineau et al., 2018), and utilizes flagellar proteins and secretion systems to enhance adhesion and immune evasion (Ghaith et al., 2018). Moreover, its strong environmental persistence enables prolonged survival and potential formation of silent transmission chains within hospitals, complicating outbreak control (Shaik Ismail et al., 2025).

Pediatric wards represent high-risk settings for healthcare-associated infections because children, especially neonates, have immature immune systems and frequently undergo invasive procedures (Shane et al., 2017; Patel and Kaufman, 2015). During blood collection, agitation and movement in infants often result in inadequate skin disinfection, contributing to contamination of blood culture specimens (Zackeroff et al., 2024; Pimentel de Araujo et al., 2017). Although *S. marcescens* is one of the Serratia species with the most abundant genomic data available (Williams et al., 2022), its potential silent circulation in pediatric units is often underestimated (Tsagris et al., 2012; Cristina et al., 2019; Bechmann et al., 2022). In cases lacking typical clinical manifestations, undetected carriers or unnoticed links in transmission chains may further obscure epidemiological investigations (Parker et al., 2023). Therefore, elucidating the dissemination characteristics of *S. marcescens* in pediatric settings is essential for strengthening early warning and infection-control strategies (Mosalli et al., 2022).

Traditional infection-control investigations rely on epidemiological tracing and environmental surveillance but often fail to resolve fine-scale genetic relationships between isolates or identify cryptic transmission events (De Silva et al., 2016; Fitzpatrick et al., 2016). Whole-Genome Sequencing (WGS) enables precise strain classification (Harch et al., 2025) and has become widely used in healthcare-associated infection source-tracking (Sundermann et al., 2022). High-resolution core-genome single-nucleotide polymorphism (SNP) analysis can reveal phylogenetic relatedness among isolates and, together with virulence gene, antimicrobial resistance gene, and plasmid profiling, allows accurate reconstruction of transmission pathways (Alam et al., 2015; Mitchell et al., 2024). Bayesian modeling and transmission-dynamic analysis can further estimate the duration of transmission and infer undetected cases (Didelot et al., 2014). The application of WGS has shifted infection-control practices from experience-based assessment to molecularly informed, high-precision identification (Brlek et al., 2024).

This study investigates a cluster of 11 *S. marcescens* bloodstream culture-positive events that occurred in the pediatric department of a tertiary hospital in 2024. By applying WGS, we aimed to differentiate true infections from contamination, clarify strain-relatedness and transmission characteristics, identify potential silent transmitters, and provide evidence to improve the interpretation of pediatric blood culture results and optimize infection-prevention strategies.

## Materials and methods

### Patient source

In October 2024, a total of 11 cases of *S. marcescens*-positive blood cultures were reported across eight pediatric wards in a tertiary hospital. Among these, three cases were identified in the Department of Pediatric Neurology, two in Pediatric Hematology, and one case each in Pediatric Cardionephrology and Endocrinology, Pediatric Respiratory Medicine Ward 1, Pediatric Respiratory Medicine Ward 2, the Department of Infectious Diseases, Pediatric Cardiothoracic Surgery, and Pediatric Orthopedics. This retrospective observational study was reviewed and approved by the hospital’s institutional ethics committee. The overall study design and analytical workflow are illustrated in Supplementary Figure 1, encompassing case collection, epidemiological investigation, laboratory testing, WGS and molecular analyses, transmission dynamics inference, as well as evaluation of intervention measures and clinical outcomes.

### Criteria for determining infection or contamination

The diagnostic criteria for hospital-acquired infections were based on the Trial Guidelines for the Diagnosis of Hospital Infections issued by the Ministry of Health of the People’s Republic of China in 2001. The classification of blood culture results as infection or contamination was performed in accordance with the 2022 Expert Consensus on the Clinical Practice of Blood Culture Techniques for the Diagnosis of Bloodstream Infections. The comprehensive assessment included: (1) the patient’s clinical manifestations and laboratory results; (2) whether the case met the diagnostic criteria for bloodstream infection; (3) whether features indicative of contamination were present, such as single-bottle positivity, lack of corresponding clinical evidence of infection, or absence of infection during follow-up. Final determinations were made jointly by at least two senior infectious disease specialists.

### Epidemiological investigation

An epidemiological investigation team was promptly established following the incident. The team comprised personnel from the Department of Hospital Infection Control, the Medical Affairs Office, the Nursing Department, the Laboratory Department, and clinical staff from the affected wards. Using the Xinglin Real-Time Hospital Infection Surveillance System (Hangzhou Xinglin Information Technology Co., Ltd., hereafter referred to as the Xinglin system), the team retrieved clinical data and relevant information for all patients with *S. marcescens*-positive blood cultures.

The investigation protocol included: (1) verification of the classification of each case as either true infection or contamination; (2) collection of demographic information and clinical data for each patient; (3) analysis of temporal, spatial, and population distribution patterns. In addition, interviews were conducted with attending physicians, nurses, specimen transport personnel, and laboratory microbiologists from the involved departments to review procedures for specimen collection, transportation, and identification, and to identify potential risk factors associated with infection or contamination.

### Environmental hygiene surveillance

Environmental sampling was conducted for all 11 *S. marcescens*-positive patients and their corresponding wards. Samples were obtained from the patients themselves, healthcare workers who had direct contact with the patients, general service staff, specimen couriers, as well as the surrounding patient environment and shared ward areas. Sampling sites included: hands of patients and other personnel; frequently touched surfaces such as bedside tables and headboard-mounted equipment; ward sinks; and in-use disinfectants. Following collection, samples were inoculated onto Columbia blood agar plates and chocolate blood agar plates (Zhengzhou Antu Bioengineering Co., Ltd.) and incubated at 35–37°C for 24–48 h. Bacterial identification was subsequently performed based on culture results.

### Isolate identification and antimicrobial susceptibility testing

Bacterial identification was performed using the Auto MS 1,000 automated matrix-assisted laser desorption/ionization time-of-flight mass spectrometry system (Zhengzhou Antu Bioengineering Co., Ltd.). Antimicrobial susceptibility testing was conducted using the Kirby-Bauer disk diffusion method, and inhibition zone diameters were interpreted according to the 2023 guidelines of the Clinical and Laboratory Standards Institute (CLSI). Results were classified as susceptible (S), intermediate (I), or resistant (R). *Escherichia coli* ATCC 25922, provided by the National Center for Clinical Laboratories under the National Health Commission, was used as the quality control strain.

### WGS and assembly of isolates

A total of 11 *S. marcescens* isolates were collected and subjected to WGS. Following genomic DNA extraction, sequencing libraries were prepared using the Illumina TruSeq DNA PCR-Free Library Prep Kit and sequenced on the Illumina NovaSeq 6000 platform (Beijing Novogene Bioinformatics Technology Co., Ltd.) with paired-end 150 bp reads (PE150). The average sequencing depth per isolate was approximately 100×. Raw reads were quality-controlled using fastp v0.23, with a quality threshold of Q30 ≥ 85% to ensure data reliability.

High-quality sequencing reads were *de novo* assembled using a complementary multi-assembler strategy. SOAPdenovo v2.04 and ABySS v2.3 were primarily used to construct draft chromosomal assemblies and optimize long-range scaffold structure from high-coverage short-read data, while SPAdes v3.15 was used to improve the assembly of repetitive regions and low-abundance plasmid contigs. The three short-read assemblies were then integrated using CISA, and the resulting scaffolds were polished with GapCloser v1.12 to fill gaps and correct local errors. For selected representative isolates, long-read sequencing data generated with the Oxford Nanopore MinION platform were incorporated, and hybrid assemblies were performed using Unicycler v0.4.9 to increase the continuity of complex genomic regions and plasmid structures.

### Analysis of antibiotic resistance genes, virulence genes, and plasmid replicons

Virulence genes in *S. marcescens* isolates were identified using the Virulence Factor Database (VFDB),^1^ while resistance genes were detected through the Comprehensive Antibiotic Resistance Database (CARD).^2^ BLAST-based screening was conducted using ABRicate v0.8.10, with gene presence confirmed when both sequence identity and coverage exceeded 80%.

Plasmid detection was performed using PlasmidFinder v2.1, with the Enterobacteriaceae database selected for scanning. A minimum identity threshold of 95% and a minimum coverage threshold of 60% were applied; replicons meeting these criteria were considered indicative of the presence of plasmids belonging to corresponding incompatibility groups.

To further characterize plasmid features, plasmid typing was conducted using MOB-suite v3.0, providing multidimensional classification data based on Replicon-MOB-Mobility profiles. These results were visualized using a heatmap to illustrate plasmid variability among isolates. Plasmid homology analysis was conducted using BRIG v0.95, which generated circular alignment maps using representative plasmids as references, thereby revealing conserved core regions and divergent variable regions. Subsequently, the igraph package was employed to construct a plasmid network map, illustrating the cross-distribution of plasmids among patients and wards and indicating potential transmission chains.

### Homology and phylogenetic analysis of isolates

Homology and phylogenetic relationships among the isolates in this study were analyzed based on SNPs. A core gene SNP-based phylogenetic tree was constructed using the maximum likelihood method to determine precise SNP distances among isolates. SNP calling was performed using Snippy v4.3, which aligned the sequences of interest to a reference dataset composed of 18 *S. marcescens* isolates from clinical blood cultures and catheters in China, obtained from GenBank. The core genome, including conserved sites, was extracted and filtered to remove recombination regions using Gubbins v2.3.4. SNP distances were calculated with snp-dists v0.7.0. Phylogenetic tree visualization and annotation were completed using the online tool ChiPlot.

### Inference of isolate transmission events

Bayesian timed phylogenetic analysis was employed to infer the transmission dynamics of *S. marcescens* isolates within the healthcare setting. Time-calibrated phylogenetic trees were constructed using BEAST v2.4.4, with core SNP multi-locus alignments from 11 isolates serving as input and sampling dates used to define temporal nodes. The optimal substitution model HKY+G (Gamma categories = 4) was selected via jModelTest. A relaxed log-normal molecular clock model and a Bayesian skyline coalescent prior were applied. The Markov Chain Monte Carlo (MCMC) was run for 100,000,000 generations with sampling every 10,000 generations. Parameter convergence was assessed using Tracer v1.7. The maximum clade credibility tree was generated with TreeAnnotator v2.4.4, with node heights indicating the median divergence time.

At the level of transmission dynamics, TransPhylo v1.4 was applied to reconstruct transmission trees based on the time-scaled phylogeny, allowing inference of the number of unsampled cases and the structure of the transmission chains. The model was configured with gamma-distributed priors for generation time and sampling delay, and posterior sampling was used to infer transmission links between detected isolates and potential cryptic cases.

## Results

### General characteristics of *S. marcescens*-positive blood cultures

In October 2024, a total of 205 blood culture samples were submitted from the pediatric department of a tertiary hospital, among which 11 isolates of *S. marcescens* were identified, yielding a detection rate of 5.37%. This rate was significantly higher than the annual detection rate in 2023 (0.35%). Among the 11 cases, 2 were classified as hospital-acquired infections, while the remaining 9 were determined to be blood culture contaminations. The cases were distributed across eight pediatric wards, including Pediatric Neurology, Pediatric Hematology, Pediatric Cardionephrology and Endocrinology, Pediatric Respiratory Medicine Wards 1 and 2, the Department of Infectious Diseases, Pediatric Cardiothoracic Surgery, and Pediatric Orthopedics, indicating widespread distribution across multiple units (Table 1).

**TABLE 1:** Clinical characteristics of 11 patients with *Serratia marcescen*s positive blood cultures.

Patient ID	Sex	Age	Department	Admission date	Date of blood culture collection	Date of isolate recovery	Strain ID	Infection/ contamination	Duration of infection (days)	Outcome
Patient 1	Female	15 years	Pediatric Neurology	1-Oct	Day 4 after admission	7-Oct	SM01	Hospital-acquired infection	7	Recovered
Patient 2	Male	5 years	Pediatric Nephro-Endocrinology	6-Oct	Day 0 (on admission)	9-Oct	SM02	Contamination	NA	NA
Patient 3	Female	0.17 years (2 months)	Pediatric Neurology	7-Oct	Day 0	10-Oct	SM03	Contamination	NA	NA
Patient 4	Male	5 years	Pediatric Neurology	7-Oct	Day 0	10-Oct	SM04	Contamination	NA	NA
Patient 5	Male	0.92 years (11 months)	Pediatric Hematology	9-Oct	Day 0	12-Oct	SM05	Contamination	NA	NA
Patient 6	Male	7 Years	Pediatric Pulmonology II	10-Oct	Day 0	13-Oct	SM06	Contamination	NA	NA
Patient 7	Male	6 years	Pediatric Hematology	11-Oct	Day 0	14-Oct	SM07	Contamination	NA	NA
Patient 8	Male	7 years	Infectious Diseases	24-Oct	Day 0	27-Oct	SM08	Contamination	NA	NA
Patient 9	Female	0.92 years (11 months)	Pediatric Cardiothoracic Surgery	20-Oct	Day 5 after admission	27-Oct	SM09	Hospital-acquired infection	5	Recovered
Patient 10	Male	6 Years	Pediatric Pulmonology I	24-Oct	Day 0	27-Oct	SM10	Contamination	NA	NA
Patient 11	Female	4 years	Pediatric Orthopedics	24-Oct	Day 3 after admission	29-Oct	SM12	Contamination	NA	NA

NA, not applicable; “Outcome” refers to the clinical resolution status for confirmed hospital-acquired infections only.

### Population distribution

Among the 11 affected patients, 7 were male and 4 were female, with ages ranging from 2 months to 15 years. For the 9 cases identified as blood culture contaminations, 8 samples were collected on the day of admission and 1 prior to elective surgery, and none of these patients exhibited clinical signs of sepsis. In contrast, the two hospital-acquired infections both developed sepsis more than 48 h after admission, and one patient required transfer to the intensive care unit due to clinical deterioration. All patients received antimicrobial therapy according to institutional protocols, with definitive treatment primarily consisting of meropenem or imipenem based on susceptibility results.

### Temporal and spatial distribution

**FIGURE 1:**
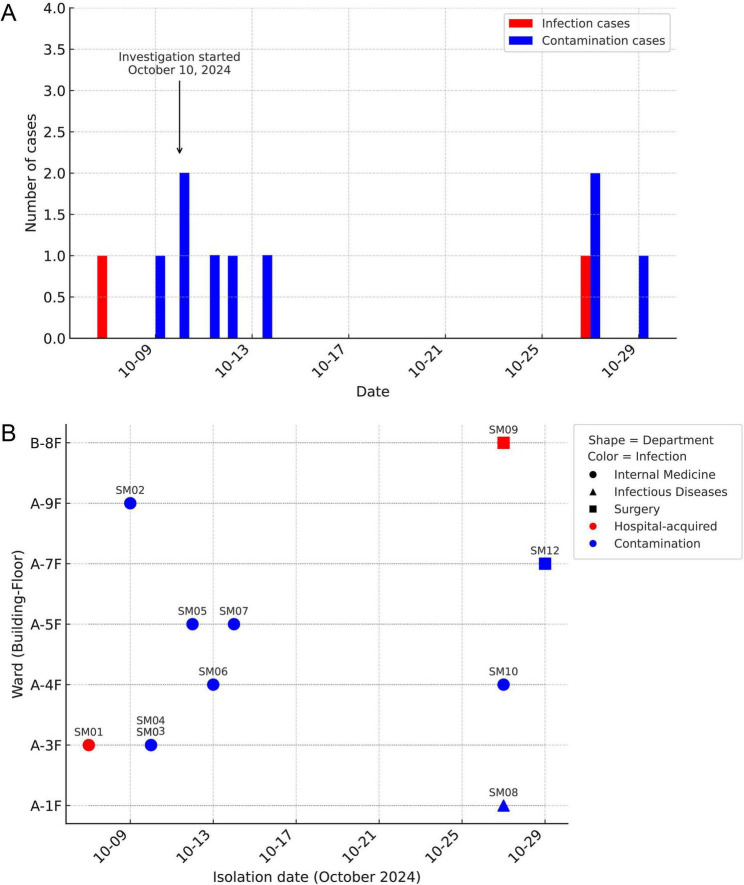
Temporal and spatial distribution of *S. marcescens* hospital-acquired sepsis and blood culture contamination. **(A)** Timeline of *S. marcescens*-positive blood cultures. Red bars indicate hospital-acquired sepsis cases; blue bars indicate contamination cases. **(B)** Spatiotemporal distribution by ward and date. Each symbol represents one isolate; different shapes correspond to department type (circle = internal medicine, triangle = infectious diseases, square = surgery), and colors denote infection classification (red = hospital-acquired, blue = contamination). The *y*-axis indicates ward location (building-floor).

Eleven cases of *S. marcescens*-positive blood cultures were primarily clustered into two outbreak periods during October 2024. The first cluster occurred within the 7-day period starting on the 7th, during which seven positive cases were reported. Significant overlap in hospitalization periods was observed, with three consecutive cases identified in the Department of Pediatric Neurology. Notably, Patient 1 and Patient 4 shared the same room with adjacent beds, indicating a potential risk of intra-ward transmission. In the following 2 days, additional cases were identified in the Pediatric Hematology Department and the second unit of the Pediatric Respiratory Department. The second cluster occurred between the 27th and 29th, with four cases reported during this interval. On the 27th alone, three cases were identified, including one confirmed hospital-acquired infection in the Department of Pediatric Cardiothoracic Surgery. On the 29th, one additional case was detected during a routine preoperative screening in the Pediatric Orthopedics Department. Overall, two distinct incidence peaks were observed in early and late October (Figure 1A).

In terms of spatial distribution, the 11 cases were predominantly located in two adjacent hospital buildings. Of these, 10 cases occurred on different floors of Building A, spanning multiple departments, including Pediatric Neurology, Pediatric Hematology, both units of Pediatric Respiratory Medicine, Pediatric Cardionephrology and Endocrinology, Infectious Diseases, and Pediatric Orthopedics. This indicates a scattered distribution across multiple wards and floors. The remaining case, a hospital-acquired infection, occurred in the Department of Pediatric Cardiothoracic Surgery located in Building B (Figure 1B). In summary, the cases exhibited two temporally distinct outbreak peaks and a spatial concentration across multiple floors of Building A, accompanied by inter-ward distribution.

### Antimicrobial susceptibility results

**FIGURE 2:**
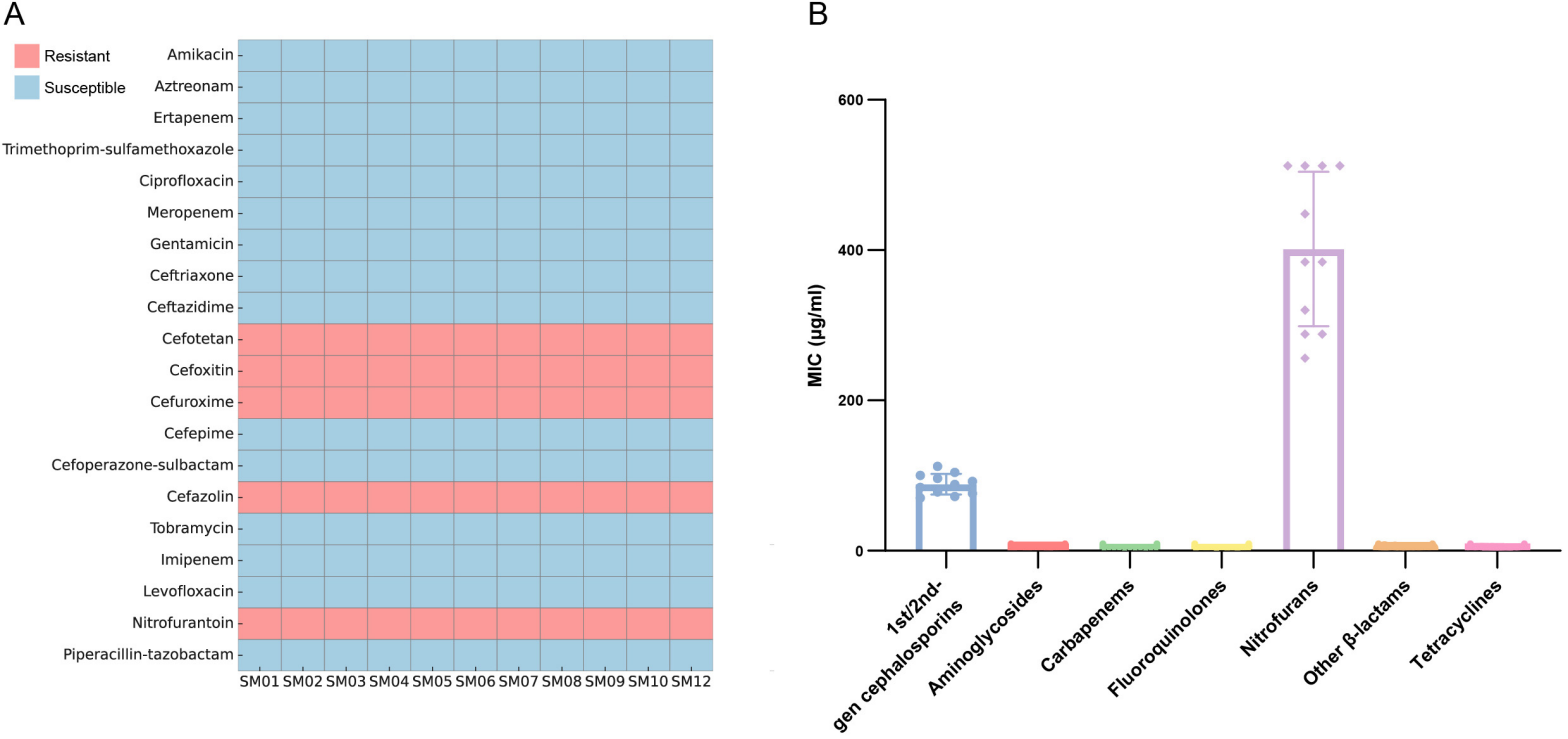
Antimicrobial resistance characteristics of clinical *S. marcescens* isolates. **(A)** Heatmap of antimicrobial susceptibility for 11 *S. marcescens* isolates. Blue indicates susceptibility; red indicates resistance. **(B)** Distribution of MICs across antimicrobial classes.

The 11 isolated strains of *S. marcescens* exhibited highly consistent resistance profiles (Figure 2A). As expected for this species, all isolates exhibited intrinsic resistance to first- and second-generation β-lactam antibiotics (e.g., cefazolin, cefotetan, cefoxitin, cefuroxime), as well as to nitrofurantoin. However, they remained susceptible to 15 other antimicrobial agents, including five additional β-lactams, three carbapenems (imipenem, meropenem, doripenem), three aminoglycosides (amikacin, gentamicin, tobramycin), two quinolones (ciprofloxacin, levofloxacin), and two tetracyclines (minocycline, doxycycline).

Further analysis of the minimum inhibitory concentration (MIC) distribution revealed uniformly elevated MIC values for first- and second-generation cephalosporins, consistent with the known intrinsic resistance of *S. marcescens* to these agents, whereas MIC values for third-generation cephalosporins and carbapenems remained low (Figure 2B). A subset of strains showed intermediate resistance tendencies to aminoglycosides, although their MIC values did not meet the threshold for formal resistance. According to the continuous antimicrobial resistance surveillance conducted in our hospital from 2019 to 2023, *S. marcescens* isolates have consistently exhibited intrinsic resistance to first- and second-generation cephalosporins, with no detection of resistance to third-generation cephalosporins or carbapenems and no evidence of ESBL- or carbapenemase-mediated mechanisms. The 11 isolates analyzed in this study showed the same susceptibility pattern, indicating that no new resistance mechanisms or increases in resistance levels have emerged in this event.

Furthermore, none of the 11 isolates displayed phenotypic features suggestive of AmpC β-lactamase derepression, and no characteristic patterns associated with AmpC induction or derepression were observed, supporting the absence of AmpC-related resistance mechanisms.

### Resistance genes, virulence genes, and plasmid profiles

**FIGURE 3:**
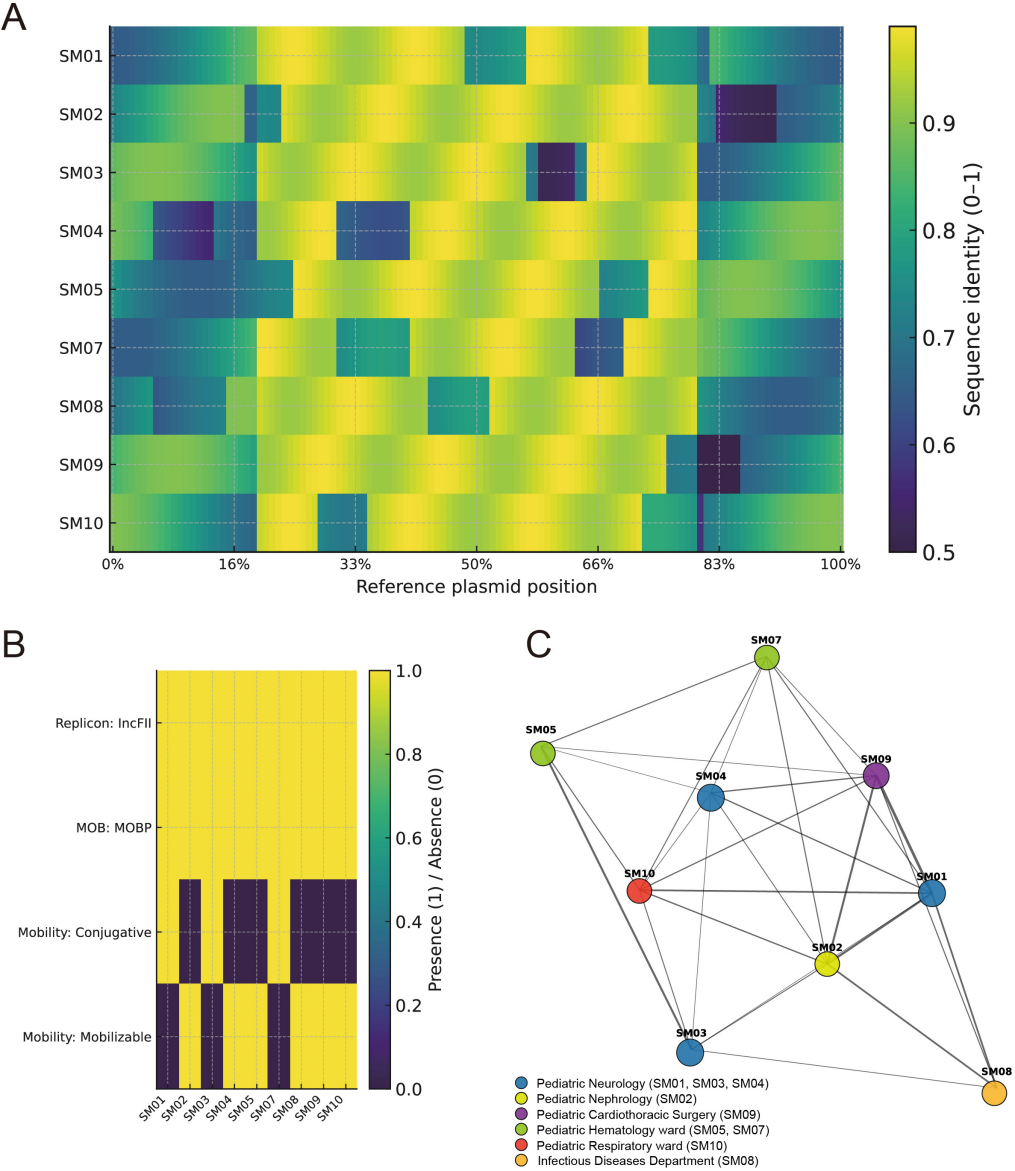
Plasmid alignment and typing analysis of *S. marcescens* isolates. **(A)** Homology heatmap based on reference plasmid sequences. Rows represent plasmid-carrying isolates; columns correspond to reference plasmid positions. The color gradient indicates sequence identity (0–1) across sliding windows. **(B)** Replicon-MOB-Mobility typing results. All plasmids carried IncFII replicons and were classified as MOBP-type. Among them, three isolates (SM01, SM03, SM07) harbored conjugative plasmids, while the remaining carried mobilizable plasmids. **(C)** Plasmid similarity network based on average nucleotide identity (ANI). Node colors indicate the wards/departments from which the isolates were obtained (Neurology, Hematology ward, Respiratory, Pediatric Nephrology, Infectious Diseases, and Pediatric Cardiothoracic Surgery), and node size is proportional to plasmid length. Edges represent plasmid similarity, with thicker lines indicating higher similarity; thick black edges denote conjugative plasmid-associated connections, whereas thin edges indicate general similarity links.

The resistance gene and virulence gene profiles of all 11 *S. marcescens* isolates were completely identical. A total of seven resistance genes were identified, including the aminoglycoside-modifying enzyme gene *aac(6’)-Ic*, the quinolone efflux pump gene *oqxB*, the streptomycin resistance gene *srt-2*, the tetracycline resistance gene *tet(41)*, and three genes associated with multidrug efflux and regulation—*mexI*, *CRP*, and *H-NS*. Regarding virulence genes, all isolates harbored *flgH*, *fliG*, *fliM*, and *cheY*, genes involved in flagellar function and chemotaxis, suggesting a potential role in adhesion, motility, and host invasion. The resistance and virulence gene profiles of 28 *S. marcescens* isolates—comprising strains from blood cultures, catheters, and this study—are shown in Figure 3A.

Among the nine *S. marcescens* isolates carrying plasmids, sequence alignment based on BRIG revealed high homology in core regions across plasmids, while variable regions exhibited deletions or divergence, indicating structural variation (Figure 3A). All plasmids carried the IncFII-type replicon and were classified as MOBP-type plasmids. Further plasmid mobility typing revealed that three isolates (SM01, SM03, SM07) harbored conjugative plasmids, while the remaining six possessed mobilizable plasmids (Figure 3B). These results suggest that although the plasmids exhibited overall structural conservation, certain variable regions differed, and the plasmids retained a limited but notable potential for horizontal transmission.

Plasmid similarity network analysis further revealed the relationships among plasmids from isolates obtained in different wards (Figure 3C). Isolates from the Department of Neurology (SM01, SM03, SM04) formed a highly homologous cluster, suggesting the presence of potential clonal transmission within this unit. SM02 originated from the Pediatric Nephrology Department, SM09 originated from the Pediatric Cardiothoracic Surgery Department, and SM08 from the Infectious Diseases Department. Isolates from the Hematology ward (SM05, SM07) also exhibited strong homology, forming a distinct subgroup. Isolates from the Respiratory Department (SM10) displayed plasmid-level similarity links to strains from both Neurology and Hematology, indicating the possible cross-ward distribution of homologous plasmids. The Infectious Diseases isolate (SM08) was also connected to multiple isolates in the network, further supporting potential inter-ward dissemination. Node size reflects plasmid length, and certain conjugative plasmids, such as those in SM01, SM03, and SM07, served as “hub nodes” connecting multiple isolates, highlighting their elevated potential for dissemination.

### Homology analysis

**FIGURE 4:**
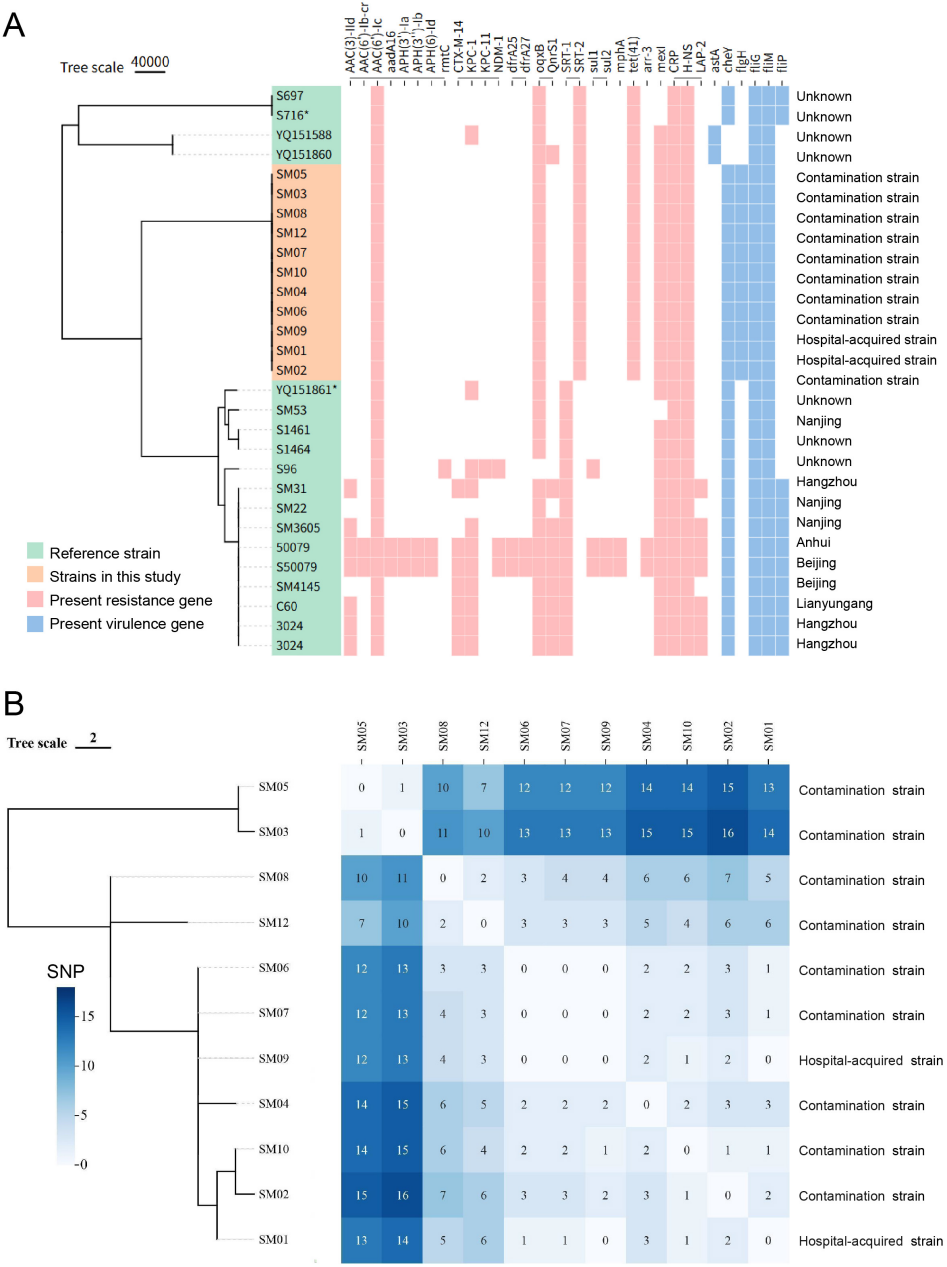
Core genome phylogenetic trees, resistance/virulence gene profiles, and SNP distance matrix of *S. marcescens* isolates. **(A)** Maximum-likelihood phylogenetic tree based on core genome alignment, including 11 *S. marcescens* isolates from this study (orange) and 17 reference strains (green). The presence of resistance genes (red) and virulence genes (blue) is shown as a heatmap to the right of the phylogenetic tree. The far-right column indicates isolate classification (hospital-acquired vs. contamination) and the geographic origin of reference strains. **(B)** SNP-based phylogenetic tree and pairwise SNP distance heatmap for the 11 isolates in this study. Color intensity reflects the number of SNP differences, as indicated by the scale bar. Hospital-acquired isolates are labeled; the others represent contamination cases. Numbers within the heatmap cells denote pairwise core-genome SNP distances between isolates, for example, SM03 and SM05 differ by only 1 SNP.

The 11 isolates in this study exhibited significant genetic divergence from 17 reference *S. marcescens* strains derived from patient blood or catheter blood in Chinese healthcare institutions, forming an independent clonal cluster (Figure 4A). Based on core genome SNP alignment, two phylogenetic trees were constructed. As shown in Figure 4B, the 11 *S. marcescens* isolates demonstrated minimal genetic variation, with a maximum SNP distance of only 16. These isolates were divided into two branches. The hospital-acquired strains SM01 and SM09 showed no SNP differences and clustered within the same branch. The contamination-related isolates SM03 and SM05 formed a distinct clonal group, with a pairwise core-genome SNP distance of only 1 SNP between them (Figure 4B). Notably, the contamination strains SM06 and SM07 shared identical SNP profiles with the hospital-acquired isolate SM09. These findings reveal a high degree of homology and a closely linked transmission chain among *S. marcescens* strains involved in this nosocomial outbreak.

### Analysis of isolate transmission events

**FIGURE 5:**
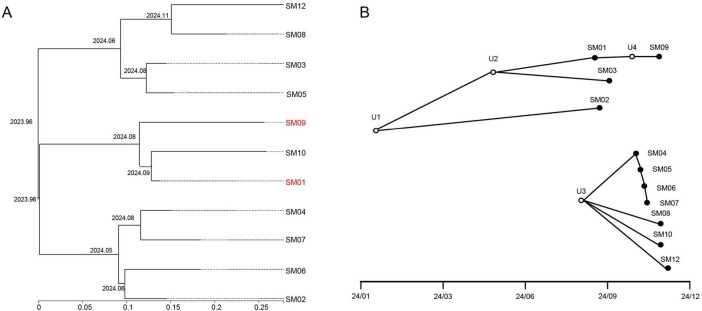
Phylogenetic and transmission inference of a *S. marcescens* bloodstream culture cluster in pediatric wards. **(A)** Time-scaled phylogenetic tree constructed using BEAST. The *x*-axis represents nucleotide differences (SNP distance), and the y-axis denotes the inferred timeline. The most recent common ancestor (TMRCA) was estimated to have emerged in late December 2023 (approximately 2023.96). Hospital-acquired isolates SM01 and SM09 are highlighted in red and clustered on the same divergence branch. **(B)** Transmission tree inferred by TransPhylo based on the BEAST-derived phylogeny. Solid circles represent sampled isolates; hollow circles indicate unsampled individuals inferred by the model (U1-U4).

Bayesian-timed phylogenetic trees revealed that the most recent common ancestor of the *S. marcescens* isolates in this study emerged in late December 2023 (2023.96). This finding indicates that the source of infection had been circulating widely in the hospital environment for at least 10 months before detection in October 2024, without effective elimination. This prolonged presence likely facilitated widespread intra-hospital transmission, ultimately leading to the observed cluster of cases. The 11 isolates diverged into three clades across three time points in 2024. The hospital-acquired strains SM01 and SM09 were located on the same divergence branch at a specific time point, despite originating from different hospital buildings. This suggests the possibility of a shared transmission chain. In contrast, blood culture contamination isolates were distributed across all three divergence points (Figure 5A).

Using TransPhylo to infer transmission events from the BEAST-derived timed phylogeny, approximately four unsampled individuals (represented as hollow nodes; 95% CrI = 1–10) were predicted in addition to the 11 sampled isolates (solid nodes) (Figure 5B). These unsampled nodes, dated between January and October 2024, served as intermediaries connecting various sampled strains. For instance, node U4 was located between SM01 and SM09, forming the path “SM01 → U4 → SM09,” which explains how these genetically related isolates could belong to the same transmission chain despite differing in collection time and hospital ward. Other unsampled nodes (e.g., U1-U3) appeared earlier, from early 2024 to the summer months, suggesting the presence of cryptic transmission before the observed cluster.

### Environmental sampling and the effectiveness of bundled infection control measures

Environmental sampling and comprehensive infection control measures were implemented concurrently with the epidemiological investigation. A total of 125 specimens were collected, including 72 hand swabs (11 from patients, 18 from physicians, 27 from nurses, 11 from ancillary staff, and 5 from specimen transport personnel), 30 samples from patients’ surroundings, 15 from ward sinks, and 8 from disinfectants in use. *S. marcescens* was not identified in any of these samples. Following a multidisciplinary meeting, bundled infection control measures were introduced, alongside interventions aimed at reducing blood culture contamination. On-site investigation revealed that pediatric ward nurses were using povidone-iodine for skin disinfection before blood collection, but did not allow sufficient drying time before puncture. Although *S. marcescens* was not detected in sink samples, a high burden of environmental microorganisms was observed. The implemented measures included: (1) replacing povidone-iodine with a faster-drying ≥ 2 g/L chlorhexidine-ethanol (70%) solution for skin disinfection; (2) replacing sinks, faucets, and pipelines in all eight wards with *S. marcescens*-positive blood cultures; (3) ensuring that healthcare workers, patients, and caregivers used alcohol-based hand sanitizers, with reinforced education, training, and supervision on hand hygiene; (4) strengthening hand hygiene monitoring for patients, caregivers, and medical staff; (5) enhancing surveillance of *S. marcescens* bloodstream infections; (6) isolating *S. marcescens* blood culture-positive patients and their caregivers in single rooms; (7) reviewing and revising the clinical specimen submission process; (8) increasing scrutiny of blood culture collection procedures by nursing staff; (9) enforcing stricter management of ward consultation physicians; and (10) upgrading terminal disinfection equipment in the wards. During the 1-month follow-up period after these interventions, no additional cases of *S. marcescens* hospital-acquired bloodstream infections or blood culture contamination were reported in the pediatric wards.

## Discussion

*S.* .*marcescens* has become an important pathogen in recent healthcare-associated infections, with multiple outbreaks reported in neonatal and intensive care settings (Aracil-Gisbert et al., 2024; Zhu et al., 2025). However, small-scale and cryptic transmission events occurring in general pediatric wards remain insufficiently characterized. In this study, we documented 11 blood culture–positive *S. marcescens* events across eight pediatric units in a tertiary hospital. Two patients developed confirmed bloodstream infections, whereas the remaining cases were initially judged as contamination; nevertheless, the isolates showed highly homogeneous genomic characteristics, indicating an underestimated clonal transmission chain. The detection rate in October 2024 (5.37%) was markedly higher than that of 2023 (0.35%), demonstrating a clear clustering pattern. Given the immature immunity of pediatric patients and the high risk of contamination during blood collection, general pediatric wards represent susceptible and easily overlooked environments. These findings underscore the need to strengthen infection surveillance in non-critical care settings.

The high number of *S. marcescens*–positive blood cultures labeled as contaminants, with only two true bloodstream infections, aligns with NICU outbreak reports where infant gut/mucosal colonization is common but invasive disease is uncommon (Montagnani et al., 2015; Mathew et al., 2015; Bourdin et al., 2023). In our cluster, the near-identical genomic and resistance profiles of all 11 isolates indicate a single hospital-adapted lineage colonizing multiple children; under such colonization pressure, difficult pediatric sampling and suboptimal povidone-iodine antisepsis likely caused inoculation into culture bottles, and the disappearance of *S. marcescens*–positive cultures after introducing chlorhexidine–alcohol skin preparation supports contamination related to colonization and technique rather than unrecognized bacteremia (Min et al., 2014; Marlowe et al., 2010).

WGS has been repeatedly shown to rapidly confirm clonality, reconstruct transmission chains, and guide infection-control interventions in NICU and ICU outbreaks (Henares et al., 2025; Merla et al., 2024). Large ICU and nationwide genomic investigations also demonstrate that *S. marcescens* can persist in hospital environments for extended periods, often without a definable point source (Zhu et al., 2025; Aracil-Gisbert et al., 2024; Taxt et al., 2025). These findings align with our BEAST/TransPhylo inference that the transmission chain likely originated in late 2023. A key distinction of this study is that the event occurred in general pediatric wards, where most cases appeared as blood culture contamination but were genomically indistinguishable. This suggests that true transmission in low-acuity settings may be more easily missed. Furthermore, compared with recent genomic studies highlighting the diversity of AmpC activity and resistance evolution (Chen et al., 2025), our isolates carried multiple resistance genes but lacked AmpC derepression mutations and remained susceptible to third-generation cephalosporins and carbapenems. This indicates an early-stage lineage with strong transmissibility but not yet advanced resistance, contributing new insights into *S. marcescens* transmission in non-critical pediatric units.

From a mechanistic standpoint, *S. marcescens* can persist in wet hospital environments such as sinks and drains, which serve as important environmental reservoirs (Aracil-Gisbert et al., 2024). Outbreaks are often associated with AmpC derepression or plasmid-mediated resistance (Chen et al., 2025). Although our isolates carried aac(6’)-Ic, oqxB, tet(41) and other resistance genes, no AmpC derepression mutations were detected, and carbapenem susceptibility was preserved—consistent with a “high transmission but low resistance” early-phase lineage. Although *S. marcescens* possesses an inducible AmpC system, WGS of our isolates revealed no ampC, ampD, or ampR mutations associated with derepression. Previous work (Pérez-Gallego et al., 2016) shows that extreme AmpC overexpression, more than 1,000-fold above wild-type levels, occurs only in amidase-deficient backgrounds and can affect resistance and fitness. No such genetic features were identified in our isolates, all of which remained susceptible to third-generation cephalosporins, thereby excluding the possibility of AmpC derepression. Additionally, the widespread presence of motility and adhesion–related virulence genes such as cheY and fliM supports enhanced environmental survival and intra-ward dissemination (Prado et al., 2022; Valiatti et al., 2023). Together, these features explain the “low virulence but high colonization and spread” phenotype observed in this event.

Although nine cases were ultimately classified as blood culture contamination, their genomic similarity to the two bloodstream infection isolates indicates that traditional contamination assessments based solely on clinical presentation and procedural evaluation may be insufficient. Previous studies have similarly reported that early, low-symptom infections during cluster events can be misclassified as contamination (Klucher et al., 2021). Integrating WGS data into contamination adjudication may therefore improve diagnostic accuracy, avoid missed infections, and prevent overlooking ongoing transmission chains.

This study has several limitations. First, the genomic analysis included only 11 *S. marcescens* isolates, which, while sufficient to define the clonal structure of this outbreak, is smaller than sample sizes typically used in large-scale WGS epidemiological investigations. Thus, the reconstructed transmission network may not capture all routes or unsampled carriers. Second, all isolates were obtained from a single pediatric center during a limited outbreak period, which may restrict the generalizability of the findings. Third, environmental sampling did not yield positive isolates, preventing assessment of whether environmental persistence contributed to the prolonged circulation suggested by the Bayesian analysis. Fourth, we did not systematically assess gastrointestinal colonization (for example, through stool or rectal screening cultures) in the nine patients with blood-culture contamination, and therefore could not evaluate the gut as a potential reservoir in this cluster. Finally, integrating WGS with broader longitudinal sampling, metagenomic surveillance, and real-time infection control assessments would further improve outbreak interpretation. Future studies with larger, multi-center datasets and continuous genomic monitoring will be crucial to refine transmission dynamics and support stronger infection control strategies in pediatric wards.

## Conclusion

**FIGURE 6:**
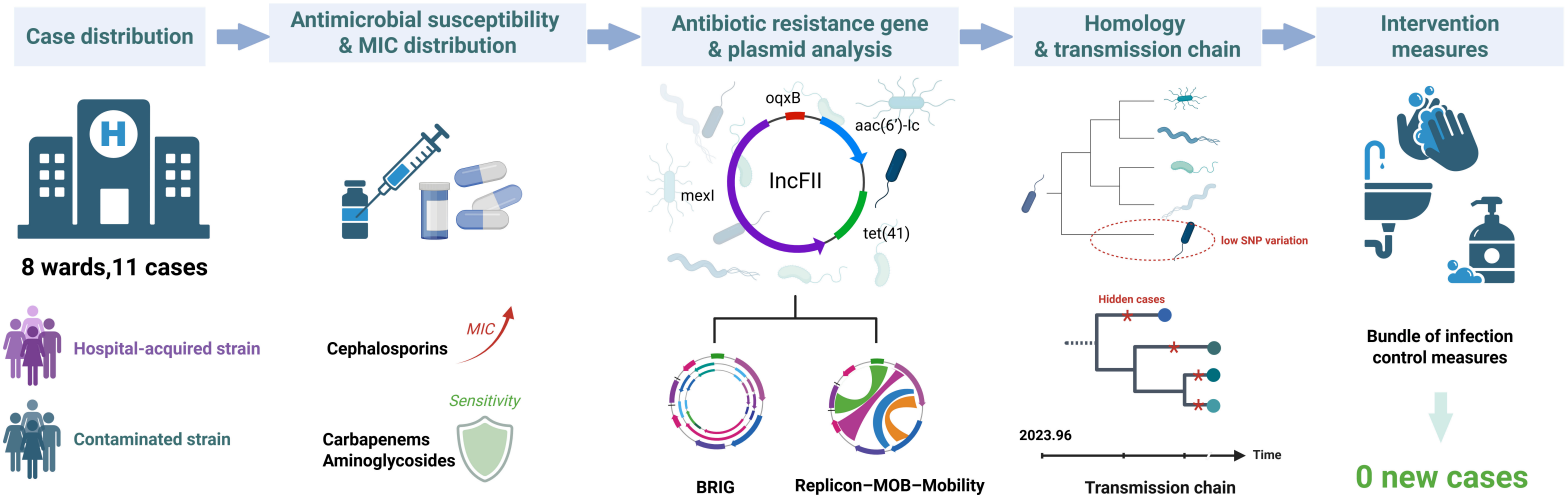
Genomic epidemiology and infection control strategies for a *S. marcescens* outbreak in a pediatric hospital.

This study reveals that a genomically uniform *S. marcescens* lineage can establish sustained transmission even within general pediatric wards, environments not traditionally viewed as conducive to long-term nosocomial persistence. Genomic reconstruction, supported by Bayesian temporal and transmission analyses, indicates that the cluster represented a prolonged and only partially detected transmission process that routine surveillance did not capture (Figure 6). The conserved resistance, virulence, and plasmid architecture suggest early plasmid-stabilized dissemination preceding the emergence of more advanced resistance traits. These findings show that low-acuity pediatric settings can maintain silent propagation of hospital-adapted organisms and illustrate the limitations of relying solely on clinical presentation to distinguish contamination from infection. More broadly, the work underscores the value of integrating real-time genomic epidemiology into pediatric infection-control frameworks to detect emerging lineages at an early stage and to intervene before transmission becomes entrenched.

## Data Availability

The original contributions presented in this study are included in this article/Supplementary material, further inquiries can be directed to the corresponding authors.

## Footnotes

1
http://www.mgc.ac.cn/VFs/main.htm

2
http://arpcard.mcmaster.ca

## Supplementary material

Supplementary Figure 1 Study flowchart.
