# Supplementary figures and images for: Whole-genome sequencing-based source tracing and infection control of Serratia marcescens blood culture events in pediatric patients
Source: Front Microbiol. 2026 Jan 16;16:1718340. doi: 10.3389/fmicb.2025.1718340 (PMC12855453; doi:10.3389/fmicb.2025.1718340)

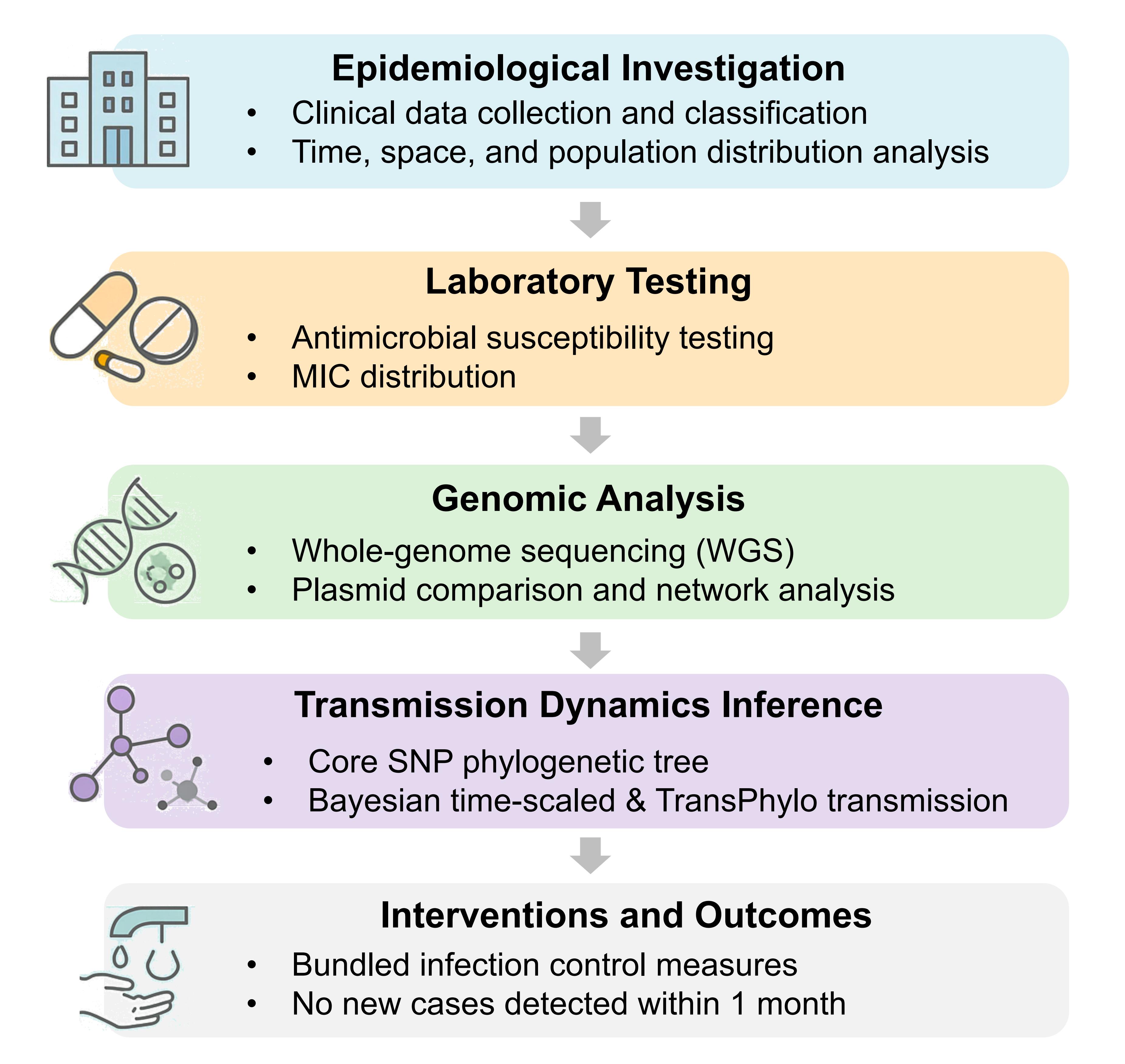

Supplement: Supplementary Figure 1 — Study flowchart. [file Image_1.jpeg]
